# Antiproliferative Potential of Cobalt(II) Phenanthroline Complexes with Pyridonates

**DOI:** 10.3390/molecules30224367

**Published:** 2025-11-12

**Authors:** Marina E. Nikiforova, Irina A. Lutsenko, Fedor M. Dolgushin, Maxim A. Shmelev, Alexey A. Sidorov, Dmitriy S. Yambulatov, Darina V. Sokolova, Vadim S. Pokrovsky, Igor L. Eremenko

**Affiliations:** 1 N.S. Kurnakov Institute of General and Inorganic Chemistry, Russian Academy of Sciences, 119991 Moscow, Russia; fmdolgushin@gmail.com (F.M.D.); shmelevma@yandex.ru (M.A.S.); sidorov@igic.ras.ru (A.A.S.); yambulatov@yandex.ru (D.S.Y.); ilerem@igic.ras.ru (I.L.E.); 2 Research Institute of Molecular and Cellular Medicine, Patrice Lumumba Peoples’ Friendship University, 117198 Moscow, Russia; d.v.sokolova@gmail.com (D.V.S.); pokrovskiy-vs@rudn.ru (V.S.P.); 3 N.N. Blokhin National Medical Research Center of Oncology, 115478 Moscow, Russia

**Keywords:** cobalt(II), 2-hydroxypyridine, 2-pyridone, pyridonate anion, 1,10-phenanthroline, complex compounds, crystal structure, cytotoxic activity

## Abstract

The reaction of CoCl_2_ · 6H_2_O with 6-chloro-2-hydroxypyridine (Hchp) and 1,10-phenanthroline (phen) afforded the complex [Co(chp)_2_(phen)] (**1**). Although this complex has been previously reported, it was obtained in this work under mild conditions (in acetonitrile at room temperature) and characterized for the first time by single-crystal X-ray diffraction. The use of Co(F_3_CCOO)_2_ · 4H_2_O under similar conditions yielded a new trinuclear molecular complex [Co_3_(chp)_2_(F_3_CCOO)_4_(phen)_2_] (**2**). According to X-ray diffraction data, the cobalt(II) ions in complexes **1** and **2** are located in an octahedral environment (coordination number CN_Co_ = 6). As an ambidentate ligand, Hchp exhibits different types of coordination modes in the resulting complexes **1** and **2**. Additional stabilization of molecules in the crystal is achieved by π-π stacking between aromatic systems of coordinated phen ligands. The cytotoxic activity of **1** and [CoCl_2_(phen)_2_] · 1.5MeCN (**3**) against a panel of human cancer cell lines (SKBR3, HCT116, A549) and normal dermal fibroblasts (HDF) was evaluated using the MTT assay. Complex **3** demonstrated cytotoxic activity against the HCT116 cell line comparable to that of cisplatin, indicating its potential as a promising antitumor agent.

## 1. Introduction

The successful introduction of cisplatin into clinical practice in 1978 stimulated an active search for new metal-based antitumor drugs that would be less toxic and more affordable. The primary focus of this research has been on coordination compounds of biogenic metals [1,2,3,4]. Copper complexes have become the most studied among them [5,6,7,8,9]. A prominent example is the well-known trade name Casiopeínas^®^, representing Cu(II) complexes with 1,10-phenanthroline or 2,2′-bipyridine and an auxiliary O,O- or N,O-donor ligand [10,11].

In contrast to copper, the antitumor properties of cobalt compounds have been studied significantly less, although recent research has shown a sustained interest in biologically active cobalt(II,III) complexes [12,13,14]. Cobalt compounds with phenanthroline are represented in the scientific literature only in small numbers [15]. Cobalt (Co) is an essential trace element that plays an important role in biological systems. The adult human body contains approximately 2 to 3 mg of cobalt, which is distributed unevenly, with the largest amounts found in the liver, bone marrow, and blood. It is also present in the thyroid gland, kidneys, and lymph nodes. The primary biological role of this transition metal is its incorporation into the structure of vitamin B_12_ (cobalamin), which participates in DNA synthesis, hematopoiesis, nervous system function, and metabolism. For normal functioning, the body requires a daily cobalt intake of approximately 20–50 µg; consumption below this range is considered a deficiency, while intake above 500 µg/day can be toxic. At such elevated levels, side effects such as oxidative stress and potential damage to cellular structures may occur [16].

Cobalt complexes exhibit diverse mechanisms of action and show significant potential as antitumor agents. Their antiproliferative effects are associated with the induction of apoptosis, inhibition of extracellular matrix-degrading enzymes, and the ability to achieve targeted delivery of cytotoxic molecules. A key advantage of these compounds stems from their redox activity, which facilitates the design of hypoxia-activated prodrugs. Such prodrugs selectively release their active payload within the hypoxic regions of tumors [15,17].

**Figure 1:**
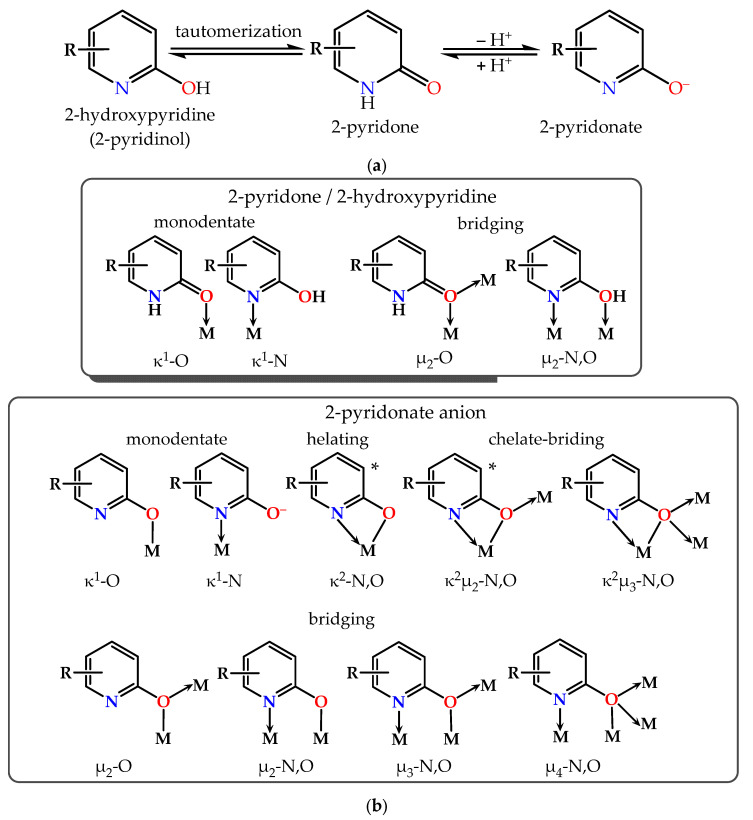
(**a**) General scheme of the tautomeric equilibrium between 2-hydroxypyridine and 2-pyridone and its deprotonation, a behavior that is also characteristic of the 6-chloro derivative; (**b**) various coordination modes of 2-pyridone/2-hydroxypyridine and its anion are known. The coordination modes of the 6-chloro-2-pyridonate anion observed in this work are depicted in the diagram *.

Pyridines and their derivatives (mono- and oligopyridines) are universal N-donor ligands that can significantly enhance biological activity through various mechanisms, such as intercalation with DNA/RNA, redox activity [18,19,20]. 2-Hydroxypyridine (2-pyridone) is used as a starting compound for the synthesis of various pharmaceutical drugs (antibiotics, anticancer and antiviral agents) [21,22]. It is a representative of triatomic bridging N,O-donor ligands. The presence of the enol fragment allows 2-hydroxypyridine and its derivatives to exist in both lactim (2-pyridinol) and lactam (2-pyridone) forms (Figure 1a) [23]. In metal complexes, the ligand can be present both in the neutral form [24,25] and in the deprotonated form (Figure 1b), forming chelate, bridged and chelate-bridged structures with different geometries—from mono- [26,27,28,29] to polynuclear homo- [29,30,31,32,33,34,35] and heterometallic [36,37,38,39] coordination compounds with various metals.

While the rich coordination chemistry of 2-hydroxypyridine (Hhp) has been well-documented for a wide range of metals, studies focusing on the cytotoxic properties of its complexes remain scarce. The few existing examples include palladium complexes, such as [PdCl_2_L_2_] (where L = 2-, 3- or 4-hydroxypyridine), which demonstrated activity against ovarian cancer cell lines [25]. However, palladium(II) and cobalt(II) centers differ fundamentally in their coordination preferences, redox behavior, and proposed mechanisms of biological action, making direct comparisons problematic. This gap in the literature regarding the cytotoxic potential of cobalt(II) complexes with 2-hydroxypyridine ligands underscores the novelty and relevance of the present study.

The aim of this work was to develop synthetic routes for obtaining cobalt(II) complexes from different starting cobalt(II) salts that would combine biologically active pyridine fragments, to determine their structures and to evaluate their antiproliferative properties against a panel of cancer cell lines, including human breast adenocarcinoma (SKBR3), human colorectal adenocarcinoma (HCT116) and human lung carcinoma (A549).

## 2. Results and Discussion

### 2.1. Synthesis and Characterization

**Scheme 1:**
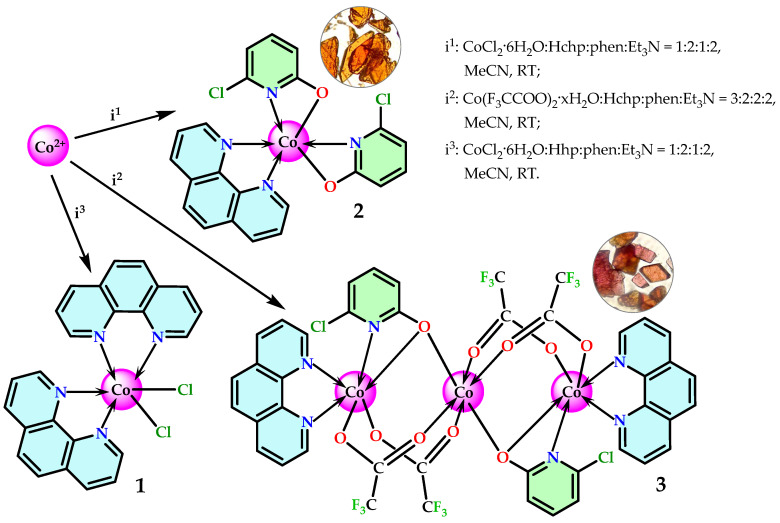
Formation of complexes **1**–**3**.

Synthesis of complexes **1** and **2** was accomplished using 6-chloro-2-hydroxypyridine (Hchp). The reaction of CoCl_2_ · 6H_2_O with Hchp in the presence of 1,10-phenanthroline (phen) and triethylamine (Et_3_N) as a deprotonating agent leads to the formation of the molecular complex [Co(chp)_2_(phen)] (**1**, Scheme 1; see also Section 3). It should be noted that the [Co(chp)_2_(phen)] complex was previously reported by Blake et al. [40]; however, its structure was not confirmed by X-ray diffraction. The compound was synthesized under harsh conditions by fusing anhydrous cobalt(II) acetate with Hchp at 160 °C under a nitrogen atmosphere, followed by recrystallization of the crude product from methylene chloride in the presence of phen. In this work, complex **1** was first obtained under mild conditions (MeCN, room temperature) and characterized by single-crystal X-ray diffraction. The use of cobalt(II) trifluoroacetate at a ratio of Co^2+^/Hchp/phen = 1:2:1 under similar conditions leads to the simultaneous crystallization of two reaction products: the previously mentioned **1** and the trinuclear heteroanionic complex [Co_3_(phen)_2_(chp)_2_(F_3_CCOO)_4_] (**2**, Scheme 1). The pure compound **2** can be obtained by carrying out the reaction with stoichiometric quantities of the reagents. The successful synthesis of **1** and **2** requires adherence to the specified sequence of reagent addition.

The use of Hhp in the reaction with CoCl_2_ · 6H_2_O showed that under mild conditions in an acetonitrile (MeCN) solution, the ligand does not react with the metal salt and does not replace chloride anions, resulting in the formation of the known complex [CoCl_2_(phen)_2_] · 1.5MeCN (**3**, Scheme 1) [41]. The metal complex [Co(phen)_2_Cl_2_], according to the literature, can be isolated both with various solvates [42,43] and in unsolvated form [44,45]. Changing the order of reagent addition in this reaction (specifically, adding Et_3_N to a solution with Co^2+^ and Hhp, followed by adding phen) does not lead to the formation of new products. After the crystallization of **3**, Hhp precipitates from the reaction system as colorless needles, this was confirmed by IR spectroscopy. The report by Sanmartin et al. [46] on the preparation of the [Co(hp)_2_(phen)] complex does not contradict our conclusion regarding the inability of hp^−^ anions to displace chloride under mild conditions, as their synthesis employed a fundamentally different method. To synthesize the cobalt complex, they performed direct electrochemical oxidation of metallic cobalt in an argon atmosphere in the presence of Hhp and phen. In this process, Hhp is reduced and deprotonated at the cathode to form the hp^−^ anion, which then coordinates with Co^2+^ ions generated during the anodic dissolution of the metal. This approach circumvents the thermodynamic limitations inherent in exchange reactions in solution. It is important to note that the work by Sanmartin et al. [46] lacks crystallographic data for [Co(hp)_2_(phen)], and its structure remains unreported in the literature. Thus, it can be concluded that in MeCN solution and in the presence of phen, the acidity of 2-hydroxypyridine is insufficient to replace chloride anions in the starting CoCl_2_. Although [Co(hp)_2_(phen)] could theoretically be obtained by the method described for the structurally characterized analog [Co(mhp)_2_(phen)] (where mhp is the 6-methyl-2-pyridonate anion) [40], our synthetic attempts were unsuccessful.

The observed difference in the reactivity of Hchp and Hhp is consistent with the influence of substituents in the pyridine ring: the electron-withdrawing Cl-substituent (–I-effect) and the electron-donating CH_3_-group (+I-effect), which is consistent with the calculated *pKa* values for Hchp and Hmhp calculated based on the Hammett equation [47,48] (8.27_Hchp_ < 11.65_Hhp_ < 12.21_Hmhp_) and is confirmed by our experimental observations. It is important to note that in the absence of competing chelating N-donor ligands, deprotonation of, for example, Hmhp by Et_3_N in MeCN proceeds successfully, leading to the substitution of nitrate and triflate anions [49]. The formation of the thermodynamically stable chelate complex [Co(phen)_2_Cl_2_] in MeCN solution shifts the overall reaction equilibrium in its favor, making the substitution of chloride ions by Hhp anions energetically unfavorable.

FTIR Spectroscopy (see Supporting Information, Figures S1 and S2).

In the IR spectrum of compound **1**, the absence of broad bands in the 3100–2800 cm^−1^ range confirms the deprotonation of Hchp; the weak, broadened vibrations in this region correspond to aromatic ν(C–H) stretching modes. The spectrum does not contain the band of the carbonyl group ν(C=O) (~1645 cm^−1^), which is characteristic for free Hchp that exists in the ketone form in the solid state. The absence of these two bands and the appearance of two intense bands at 1595 cm^−1^ (ν_as_(C–O)) and 1453 cm^−1^ (ν_s_(C–O)) indicate that Hchp in complex **1** exists in the anionic form. In the 1530–1340 cm^−1^ region, corresponding to the skeletal vibrations of the aromatic rings of the ligands (ν(C=C) and ν(C=N)), significant changes are observed compared to free Hchp and phen, which indicate their coordination. Further in the low-frequency region, deformation vibrations δ(C–H) of the ligands at 837 cm^−1^ and a band at 724 cm^−1^, characteristic of ν(C–Cl), are observed [48,50,51,52].

The IR spectrum of complex **2** also lacks a band of the carbonyl group ν(C=O) and contains two intense bands at 1589 cm^−1^ (ν_as_(C–O)) and 1438 cm^−1^ (νs(C–O)). In the spectrum, the ν_as_(COO) and ν_s_(COO) bands, characteristic of the trifluoroacetate anion, are shifted to the high-frequency region (1686 cm^−1^) and the low-frequency region, respectively (1438 cm^−1^), compared to those in Co(F_3_CCOO)_2_ [53]. Further, bands characteristic of the skeletal vibrations of aromatic rings (ν(C=C) and ν(C=N)), deformation vibrations δ(C-H) at 843 cm^−1^ of the chp^−^ and phen and bands at 722 cm^−1^ and 699 cm^−1^, corresponding to ν(C-F) and ν(C-Cl), are observed.

According to the XRPD data for **1** and **2** in the 2θ range from 5 to 45 degrees (see Supporting Information, Figures S3 and S4), the samples are single-phased. The purity of compound **3** was also confirmed by XRPD data (Figure S5).

### 2.2. Crystal Structure

#### 2.2.1. Crystal Structure of **1**

**Figure 2:**
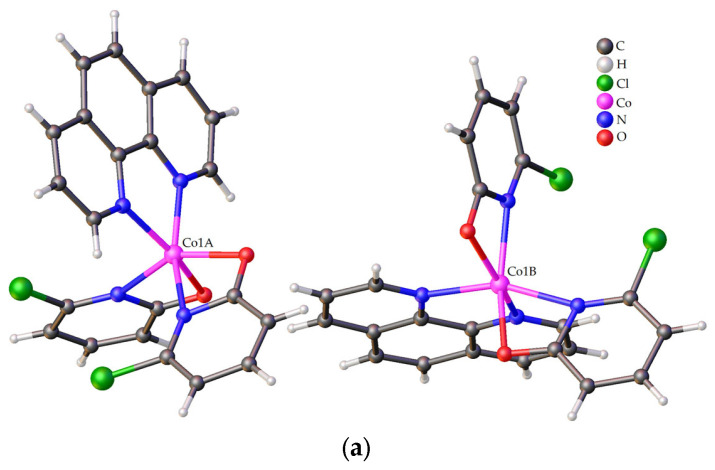

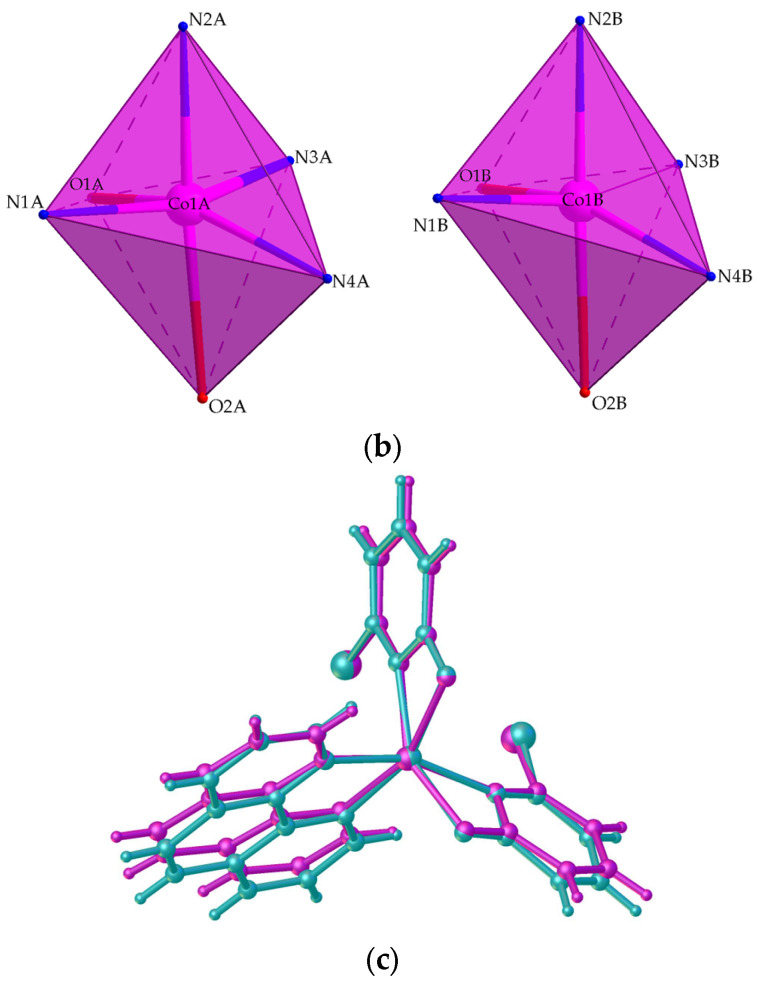
(**a**) Arrangement of independent molecules of compound **1** in the crystal; (**b**) structure of Co1A and Co1B coordination polyhedra; (**c**) overlap images of independent molecules belonging to Co1A (pink) and Co1B (cyan) relative to two four-membered chelate rings of the 2-pyridonate anions.

According to X-ray diffraction data, compound **1** crystallizes in the triclinic space group *P*-1 in the form of two independent molecules [Co(chp)_2_(phen)] of the same structure (Figure 2a, Table 1 and Table S1). The coordination environment of both cobalt(II) atoms is six-coordinate (Figure 2b,c). CShM calculations performed using the SHAPE 2.1 program [54,55,56] confirmed a highly distorted octahedral coordination geometry of cobalt atom (OC-6, *O*_h_). Table 2 presents the calculated CShM values, which allow us to quantitatively assess the degree of deviation of the studied structures from ideal polyhedra.

For the independent molecule with the metal cation Co1B, the distortion is more pronounced (*S*_q_ = 5.030) than for the molecule with the metal cation Co1A (*S*_q_ = 4.819). The next closest polyhedral models are the trigonal prism (TPR-6, *D*_3h_), with CShM values are 9.638 and 8.918, respectively. The distortion of the coordination environment of cobalt is determined by the formation of strained four-membered chelate metallocycles during the coordination of chp^−^ anions, in which the angle at the metal atom is on average 62.7°. Similar distortions of the octahedral environment around the cobalt ion have been reported for the related complex [Co(mhp)_2_(phen)] [40].

**Figure 3:**
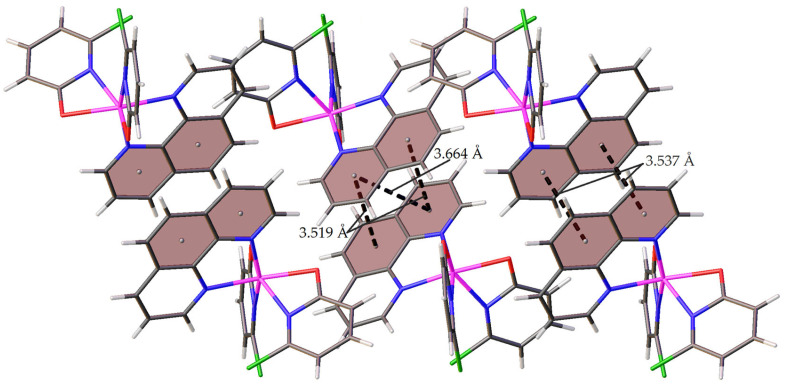
Intermolecular π-π interactions between molecules **1** in the crystal.

**Figure 4:**
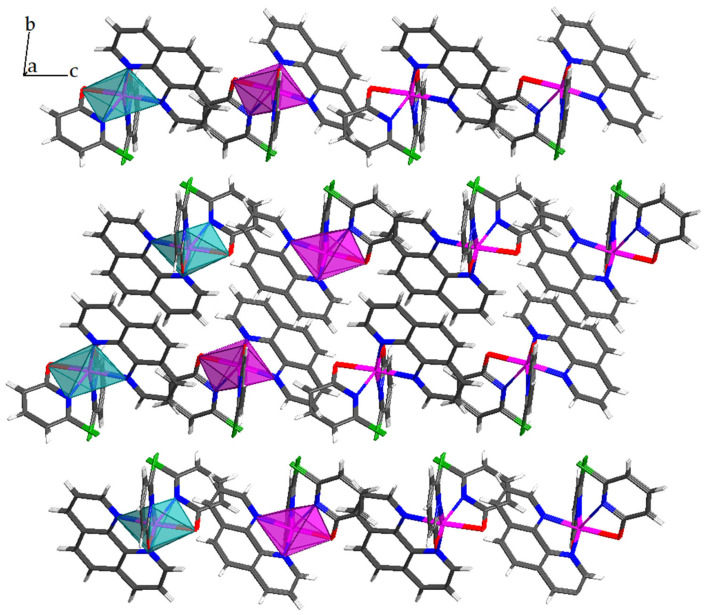
Crystal packing **1** in projection onto the *bc* plane, the coordination polyhedra of Co1A ions are highlighted in pink, and Co1B ions in cyan.

In the crystal packing of compound **1**, the aromatic rings of the N-donor ligand participate in intermolecular π-π interactions with the rings of another independent molecule—the distances between the centroids of the interacting fragments and the angle between the planes are 3.537 Å at 1.652° and 3.519 Å at 2.372°, respectively (Figure 3, Table S2). This interaction promotes the sequential arrangement of independent molecules in the crystal with the formation of supramolecular chains, which in turn are oriented towards each other in the crystal by chlorine atoms (Figure 4).

#### 2.2.2. Crystal Structure of **2**

**Figure 5:**
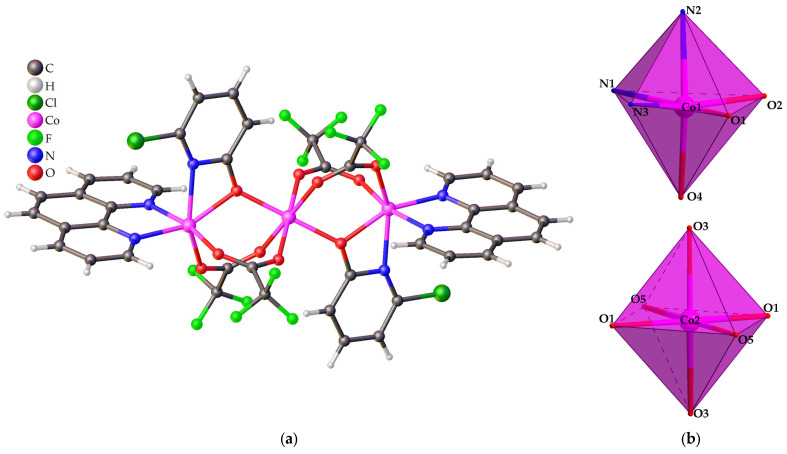
(**a**) The molecular structure of complex **2** in a crystal; (**b**) the coordination environment of Co1 and Co2.

The trinuclear heteroanionic complex **2** (Figure 5a, Table 1 and Table S1) crystallizes in the triclinic space group *P*-1. The molecule is centrosymmetric with respect to the inversion center passing through the central Co(II) ion. Two cobalt atoms are linked by two bridging trifluoroacetate and one chelating-bridging 6-chloro-2-pyridonate anions. In this case, the polyhedron of the central cobalt(II) ion is an almost ideal octahedron *O*_h_, consisting only of oxygen atoms CoO_6_ (Figure 5b). This is confirmed by the calculated CShM value for Co2 equal to 0.090 (see Table 2). The oxygen atoms of trifluoroacetate anions are located at the base of the coordination polyhedron, and the oxygen atoms of 2-pyridonate anions are located at the apex of the pyramid. The terminal cobalt(II) ions Co1 in **2** are located in a distorted octahedral CoN_3_O_3_ environment (Figure 5b), for which the CShM value is 3.240. At the base of the bipyramid there are two oxygen atoms from the 2-pyridonate and trifluoroacetate anions and two nitrogen atoms from the 2-pyridonate anion and the coordinated phen molecule. At the vertices of the bipyramid are located the oxygen atoms of the trifluoroacetate anion and the nitrogen atoms of the phen. Such a trinuclear fragment is typical for 3*d*-metal carboxylates, where the position of the 2-pyridonate anion is occupied by other bridging or chelate-bridging anions [57,58,59,60,61,62,63].

**Figure 6:**
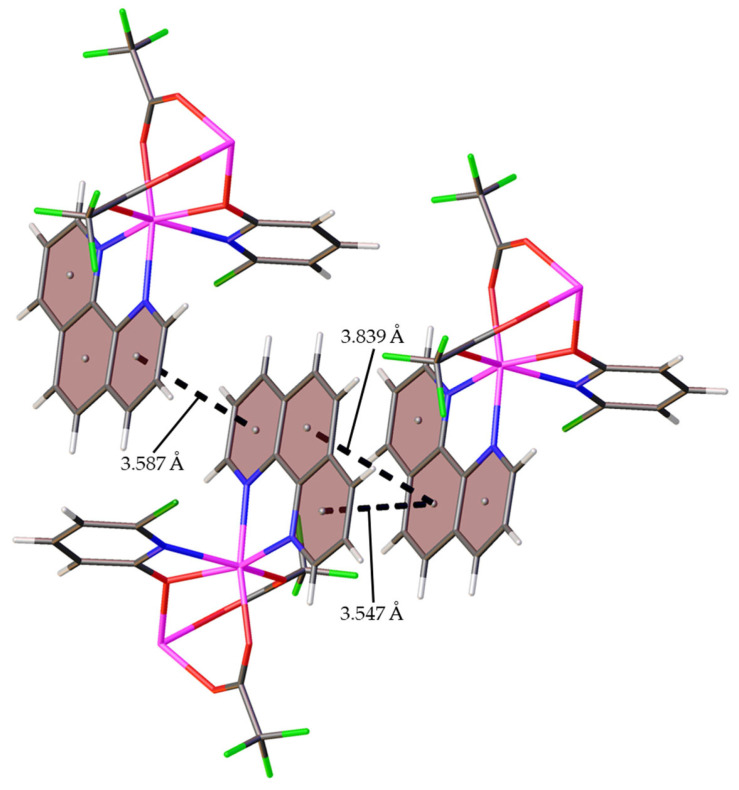
A fragment of the crystal packing in a crystal of **2**, illustrating π-π intermolecular interactions between aromatic rings of phen (highlighted in pink; for clarity, only the asymmetric unit of the molecules are shown).

**Figure 7:**
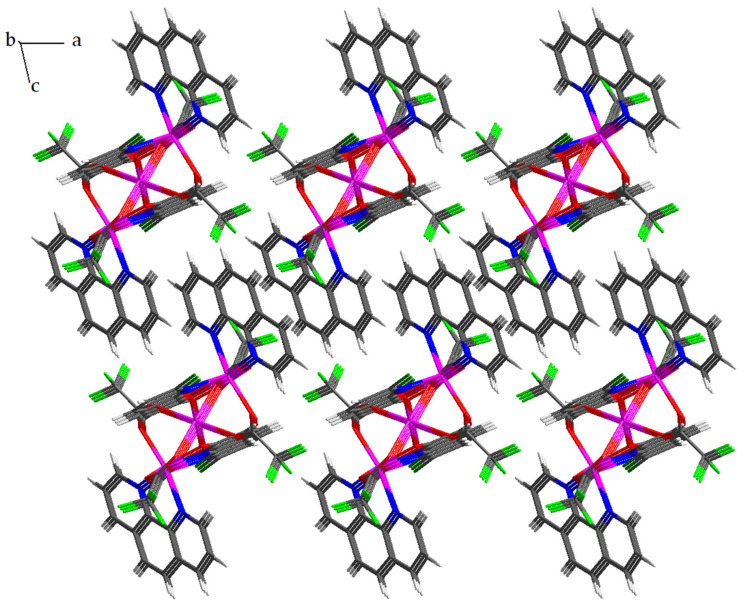
Crystal packing of **2** in projection onto the *ac* plane.

The molecules of complex **2** in the crystal are arranged in a checkerboard pattern, and π-stacking interactions are observed between the coordinated phen molecules. The distances between the centroids of the interacting fragments and the angle between the planes are equal to 3.547 Å and 0.834° and 3.587 Å and 1.501°, respectively (Figure 6, Table S2). This arrangement of **2** molecules in the crystal in the *ac* plane forms supramolecular rows of trinuclear metal cores and coordinated molecules of the N-donor ligand (Figure 7).

### 2.3. UV/Vis Absorption of Compound ***1***–***3***

**Figure 8:**
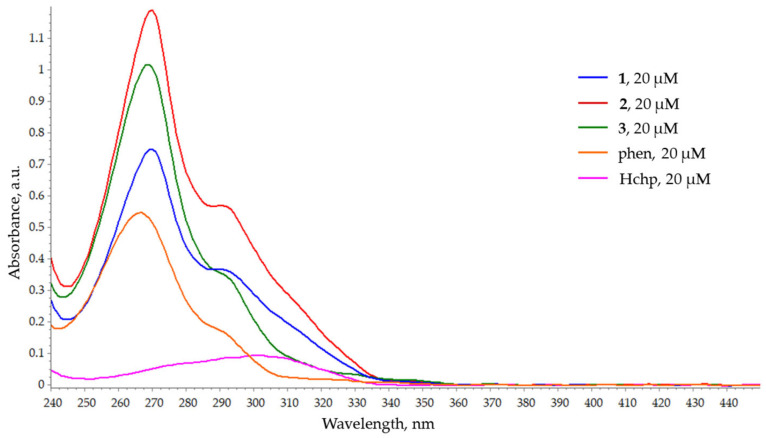
UV–visible absorption spectra of complexes **1**–**3** in a 1% aqueous DMSO solution immediately after preparation (0 h).

**Figure 9:**
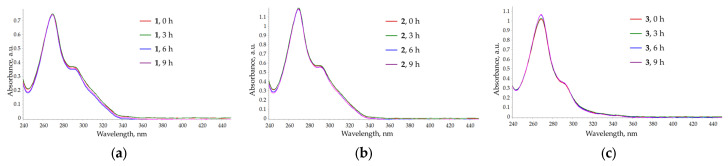
Time-dependent changes in the UV–visible absorption spectra of a 20 µM solution of complexes **1** (**a**), **2** (**b**), and **3** (**c**) in a 1% aqueous DMSO solution.

The absorption spectra of complexes **1**–**3** in the UV–visible region were recorded in the 240–500 nm range in a 1% aqueous DMSO solution immediately after preparation (0 h) (Figure 8) and at three-hour intervals (Figure 9). A comparison of the spectra obtained at different time points revealed no significant changes in the position, shape, or intensity of the absorption bands for any of the investigated complexes over time (Figure 9). The absence of any band shifts or changes in their optical density indicates that the structure of the complexes remains unchanged. This observation provides direct evidence for the stability of the solutions of these complexes under the applied conditions (22 °C) for at least 9 h.

The molar extinction coefficients (ε) of complexes **1**–**3** were calculated in accordance with the Beer–Lambert–Bouguer law (*A* =*εbc*) from calibration plots constructed in the UV-Vis region. A series of solutions of varying concentrations, prepared by serial dilution of a 100 μM stock solution, was used for this purpose (see Supplementary Materials, Figures S6–S8). The calculated ε values were 39,200 L·mol^−1^·cm^−1^ (R^2^ = 0.999) for complex **1**, 59,700 L·mol^−1^·cm^−1^ (R^2^ = 0.9998) for complex **2**, and 42,900 L·mol^−1^·cm^−1^ (R^2^ = 0.983) for complex **3**. The values of the coefficient of determination (R^2^) confirm the adherence to the Beer–Lambert–Bouguer law and the linear dependence of the optical density on concentration within the studied range.

### 2.4. Cytotoxic Properties of ***1*** and ***3***

**Figure 10:**
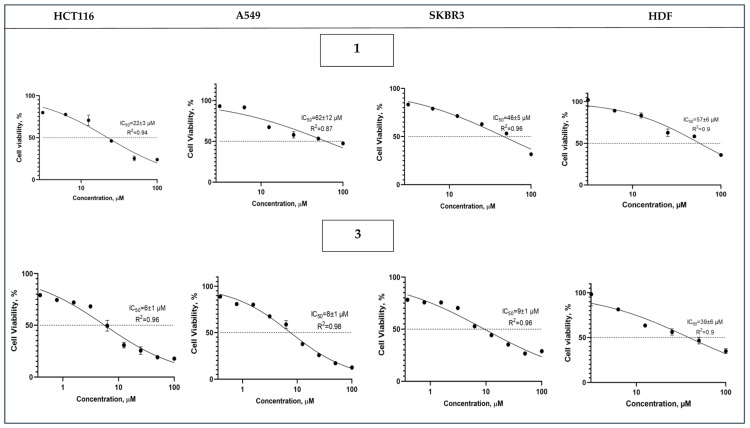
Survival of HCT116, A549 and SKBR3 cells incubated with complexes **1** and **3**. Co-incubation time 72 h.

Based on the results obtained during studies of the cytotoxic activity of complexes **1** and **3** in relation to the HCT116, A549 and SKBR3 cell lines, pharmacodynamic dose-effect curves were constructed (Figure 10), as well as average mean values of the application of concentrations causing 50% inhibition of cell survival (50% inhibitory concentration—IC_50_) (Table 3).

Both complexes exhibited a pronounced cytotoxic effect against a panel of studied human tumor cell lines of various histogenesis with IC_50_ values in the micromolar range. However, complex **3** demonstrated higher activity compared to complex **1**, as evidenced by the obtained calculated IC_50_ values—4–8 times lower compared to the corresponding values for complex **1**. A comparative assessment of the cytotoxic activity of both complexes with the reference drug cisplatin showed that complex **3** demonstrated a comparable level of activity against the HCT116 cell line (IC_50_ = 6 ± 1 µM (**3**) and 8 ± 0.5 µM (cisplatin), *p* < 0.05). For comparison, Table 3 provides data for other compounds containing gold or europium phenanthroline fragments tested on similar cell lines [64,65]. Although these gold and europium complexes show higher potency, compound **3** exhibits superior selectivity. It should also be noted that compound **3** exhibited moderate selectivity towards tumor cells compared to cytotoxic activity towards normal fibroblasts from a healthy donor, which served as a healthy control in our studies—SI = 4.1–6.8. This allows us to consider this complex as promising for the treatment of colorectal cancer and to proceed with an in-depth preclinical study of this complex, exploring the molecular mechanisms of its action. It should also be noted that the presumably higher stability of [Co(chp)_2_(phen)] in biological media compared to [CoCl_2_(phen)_2_] could be beneficial for medicinal applications. Despite its apparently lower activity, this enhanced stability may allow for a more sustained delivery of the phen ligand to cancer tissues.

## 3. Materials and Methods

### 3.1. General Remarks

Commercially available reagents were used for the synthesis without additional purification: 2-hydroxypyridine (98%), 6-chloro-2-hydroxypyridine (98%), CoCl_2_ · 6H_2_O (99%), 1,10-phenanthroline monohydrate (99%), triethylamine (99%), acetonitrile (≥99%), F_3_CCOOH (99%) and Co_5_(CO_3_)_2_(OH)_6_ (99%). Co(F_3_CCOO)_2_ · 4H_2_O was prepared by an exchange reaction of excess cobalt(II) carbonate hydroxide with F_3_CCOOH. Complex **3** was synthesized according to [41] in air, and its purity was verified by X-ray powder diffraction (XRPD).

Elemental analysis was performed on an automatic C,H,N,S analyzer EuroVector EA3000 (Euro-Vector, Pavia, Italy). The IR spectra of the compounds were recorded on a Spectrum 65 spectrometer (Perkin Elmer, Waltham, MA, USA) equipped with a Quest ATR Accessory with a diamond top-plate assembly (Specac, Orpington, Kent, UK) attenuated total internal reflection attachment. The spectra were obtained in the range 400–4000 cm^−1^ with a spectral resolution of 8 cm^−1^ and an accumulation of 20 scans.

X-ray powder diffraction (XRPD) patterns were recorded on a Bruker D8 Advance diffractometer (Bruker AXS GmbH, Karlsruhe, Germany) using CuKα radiation (λ = 1.5406 Å, Ni filter, LYNXEYE detector, reflection geometry) within a 2θ range of 5–45 degree and with a signal collection time of 2 s per step.

Optical absorption spectra of solutions of the starting reagents and the resulting complexes were recorded in a 240–450 nm range, at 1 nm steps on an SF-2000 spectrophotometer (OKB Spectr, Saint-Petersburg, Russia).

The XRD study of coordination compounds **1** and **2** was conducted at 100 K on a Bruker D8 Venture diffractometer (Bruker AXS GmbH, Karlsruhe, Germany; CCD detector, MoKα, λ = 0.71073 Å, ω-scanning). A semi-empirical absorption correction for all the compounds was calculated with the SADABS-2016/2 program [66]. The structures were solved by direct methods and refined by full matrix least squares on F^2^ with anisotropic thermal parameters for all non-hydrogen atoms using the SHELXL-2018/3 program [67]. The positions of hydrogen atoms were calculated geometrically and refined in the isotropic approximation using the “rider” model with Uiso(H) = 1.2Ueq(C). In structure **2**, the fluorine atoms of one of the trifluoroacetate groups are disordered over two positions with nonequivalent occupancies of 0.635(5)/0.365(5), which were refined using standard constraints (DFIX, EADP, ISOR). The crystallographic parameters and the structure refinement statistics are shown in Table 4. Supplementary crystallographic data for the compounds synthesized are given in CCDC numbers (2483770 and 2483771). These data can be obtained free of charge from the Cambridge Crystallographic Data Centre via www.ccdc.cam.ac.uk/data_request/cif (accessed on 13 October 2025).

### 3.2. Biological Activity: Cell Culture and Cytotoxicity Assays

A panel of human solid tumor cell lines was used in the in vitro study: breast adenocarcinoma (SKBR3, HTB-30), colorectal adenocarcinoma (HCT116, CCL-247), and lung carcinoma (A549, CCL-185) were obtained from the American Type Culture Collection (ATCC, Rockville, MD, USA) with short tandem repeat-based authentications and mycoplasma-free status. Cytotoxic selectivity was evaluated using a non-tumor culture of human dermal fibroblasts (HDF, Lonza, CC-2511). Cells were grown in RPMI-1640 medium (Roswell Park Institute) supplied with 10% FBS (fetal bovine serum, HyClone/Cytiva, Marlborough, MA, USA), 1% penicillin/streptomycin and incubated at 37 °C with 5% CO_2_. The cells were seeded in a 96-well sterile plate (3–6 × 10^3^ cells/well) and kept overnight at 37 °C in 95% humidity supplied with 5% CO_2_. Series of different concentrations of compounds were made with DMSO and serum-free medium and added to the cells. The maximum concentration of DMSO in the investigations was <0.1% and demonstrated no cell lethality. The cells were co-incubated with the compounds for 72 h. Next, 20 µL of the MTT staining reagent (5 mg/mL in phosphate cradle arrangement) was added to each well and the plates were incubated at 37 °C. After 4 h of incubation the supernatant was removed and 200 µL of dimethyl sulfoxide (DMSO) was added to each well to solubilize the formazan crystals, and absorbance was recorded with a 540 nm using microplate reader (Multiscan FC, Thermo Fisher Scientific, Waltham, MA, USA). Untreated cells were run in each assay as the negative control group. All the experiments were performed in triplicate. The IC_50_ values were calculated by a nonlinear regression curve using GraphPad Prism version 9.0 for Windows. IC_50_ values are presented as mean ± standard deviation (SD). The results were analyzed using one-way ANOVA with Tukey’s multiply comparison test using SPSS 21 software and were accepted as significantly different when *p* < 0.05.

### 3.3. Synthesis of ***1***

Cobalt(II) chloride CoCl_2_ · 6H_2_O (0.119 g, 0.50 mmol) and 6-chloro-2-hydroxypyridine (0.129 g, 1.00 mmol) were dissolved in 15 mL of MeCN. A sample of phen (0.099 g, 0.50 mmol) was added to the blue solution, after which the color of the solution became green-blue, to which triethylamine (0.14 mL, 1.00 mmol) was then added. Orange crystals suitable for X-ray diffraction begin to precipitate from the resulting green solution after ~10 min. The resulting crystals were separated from the mother liquor by decantation, washed with cold (−18 °C) MeCN and dried under ambient conditions. The yield was 0.218 g (87% based on consumed Hchp). Anal. calc. for C_22_H_14_Cl_2_N_4_O_2_Co: C 53.25, H 2.84, N 11.29 Found: C 53.45, H 2.67, N 11.44. FT-IR (ATR, ν/cm^−1^): 3071 w, 3054 w, 3013 w, 1595 s, 1585 s, 1527 m, 1516 m, 1453 s, 1422 s, 1403 s, 1355 m, 1342 m, 1248 m, 1223 m, 1159 s, 1138 m, 1101 m, 1057 w, 987 s, 919 s, 868 w, 851 s, 775 s, 730 s, 695 s, 642 m, 567 m, 529 m, 452 w, 424 m.

### 3.4. Synthesis of ***2***

Cobalt(II) trifluoroacetate Co(F_3_CCOO)_2_ · 4H_2_O (0.268 g, 0.75 mmol) and 6-chloro-2-hydroxypyridine (0.0648 g, 0.50 mmol) were dissolved in 15 mL of MeCN. Phen (0.099 g, 0.50 mmol) was added to a red-orange solution. Triethylamine (0.07 mL, 0.50 mmol) was then added to the reaction mixture. After a few minutes small pink crystals begin to fall out of the resulting pink solution. The resulting crystals were separated from the mother liquor by decantation, washed with cold (−18 °C) MeCN and dried under ambient conditions. Crystals suitable for X-ray diffraction were obtained by slow evaporation of the mother liquor decanted from the reaction product. The yield was 0.243 g (77% based on consumed Hchp). Anal. calc. for C_42_H_22_Cl_2_F_12_N_6_O_10_Co_3_: C 40.47, H 1.78, N 6.74 Found: C 40.24, H 1.71, N 6.89. FT-IR (ATR, ν/cm^−1^): 1686 s, 1589 s, 1543 w, 1518 w, 1438 s, 1341 m, 1191 s, 1171 s, 1138 s, 994 m, 940 m, 843 s, 793 s, 775 m, 722 s, 699 m, 643 m, 626 w, 613 w, 549 m, 528 m. 455 m, 425 w.

## 4. Conclusions

Mono- **1** and trinuclear **2** cobalt(II) complexes were obtained using Hchp and phen under mild conditions. X-ray structural analysis revealed an octahedral environment of the cobalt(II) atom in the resulting coordination compounds. In mononuclear complex **1**, two chp^−^ anions form strained four-membered metallocycles, distorting the polyhedron. Additional stabilization of the crystal structures of **1** and **2** is provided by intermolecular π-π interactions of the aromatic rings of the phen ligands. This study also revealed a key difference in the reactivity of 6-chloro-2-hydroxypyridine and unsubstituted 2-hydroxypyridine in the presence of phen in MeCN solution, due to differences in their acidity. The cytotoxicity of complexes **1** and **3** was studied on the tumor cell lines HCT116, SKBR3, and A549. Complex **3** exhibited higher activity (IC_50_ 6–9 μM) compared to **1** (IC_50_ 22–62 μM), as well as moderate selectivity for tumor cells compared to normal skin fibroblasts (SI 4.1–6.8), while **1** did not show such selectivity (SI 0.9–2.6). The data obtained, particularly the high activity and selectivity of complex **3** against human colorectal adenocarcinoma, suggest it is a promising compound for in-depth preclinical studies, including investigation of the molecular mechanisms of its antitumor action.

## Figures and Tables

**Table 1:** Principal bond lengths (Å) for compounds **1** and **2**.

Bond	*d*, Å	
	1	2
Co–O(chp)	2.0963(15) 2.1619(15) 2.0988(16) 2.1679(15)	2.027(3) 2.092(3)
Co–N(chp)	2.1209(17) 2.1819(17) 2.1233(17) 2.1698(18)	2.250(4)
Co–N(η-phen)	2.1201(19) 2.1091(17) 2.1136(18) 2.1245(18)	2.091(4) 2.130(4)
Co–O(µ-OOCCF_3_)	―	2.087(3) 2.060(3) 2.103(3) 2.114(3)

**Table 2:** Parameters describing distortion in the coordination polyhedra of compounds **1** and **2**.

Compound		OC-6 ^1^	TPR-6 ^2^	PPY-6 ^3^
**1**	Co1A	4.819	9.638	21.427
	Co1B	5.030	8.918	20.965
**2**	Co1	0.090	16.222	29.264
	Co2	3.240	9.593	21.455

^1^ OC-6—octahedron *O*_h_; ^2^ TPR-6—trigonal prism *D*_3h_; ^3^ PPY-6—pentagonal pyramid *C*_5v_.

**Table 3:** Cytotoxicity (IC_50_ ± SD µM) of the compounds against human solid cell lines and normal fibroblasts for 72 h.

Compound	IC_50_, µM				SI ^a^			Ref.
	HCT116	A549	SKBR3	HDF	HCT116	A549	SKBR3	
**1**	22 ± 3	62 ± 12	46 ± 5	57 ± 6	2.6	0.9	1.2	This work
**3**	6 ± 1 *	8 ± 1	9 ± 1	39 ± 6	6.8	5.1	4.1	This work
(phenH_2_)[AuCl_4_]∙NO_3_	88.9 ± 3.2	132.3 ± 45.4	112.5 ± 10.7	228.3 ± 13.9	2.6	1.7	2.0	[64]
[Au(neoc)Cl_3_] ^b^	0.2 ± 0.05	2.60 ± 1.18	2.00 ± 0.56	35.62 ± 2.45	169.6	13.7	17.8	[64]
[Eu_2_(tph)_4_(OAc)_2_(phen)_2_] ^c^	3.7 ± 0.4	4.0 ± 0.7	8.2 ± 1.2	7.3 ± 1.4	3.0	1.9	1.8	[65]
Cisplatin ^d^	8 ± 0.5	5 ± 0.3	8 ± 0.6	23 ± 2	2.9	4.6	2.9	This work

^a^ SI—selectivity index of the cytotoxic agent, calculated using the formula: IC_50_ (healthy donor fibroblasts)/IC_50_ (tumor cells); ^b^ neoc—neocuproine; ^c^ tph and OAc—thiophencarboxylic and acetic acid anions, respectively; ^d^ Data on the cytotoxicity of the reference drug cisplatin was obtained in our previous study; * *p* < 0.05.

**Table 4:** Selected crystal data and parameters for structure refinement of **1** and **2**.

Parameters	Complex	
	1	2
Empirical formula	C_22_H_14_Cl_2_CoN_4_O_2_	C_42_H_22_Cl_2_Co_3_F_12_N_6_O_10_
Formula weight	496.20	1246.34
Crystal system	Triclinic	Triclinic
Space group	*P*-1	*P*-1
*a* (Å)	9.3330 (5)	9.7217 (8)
*b* (Å)	14.7266 (7)	11.0191 (9)
*c* (Å)	15.0683 (7)	12.0031 (11)
α (°)	82.8863 (16)	69.720 (3)
β (°)	89.2581 (17)	76.610 (3)
γ (°)	81.6392 (16)	87.115 (3)
V (Å^3^)	2033.23 (17)	1172.70 (18)
Z	4	1
*D*_calc_ (g∙cm^−3^)	1.621	1.765
Temperature (K)	100	100
*μ* (mm^−1^)	1.135 (Mo-Kα)	1.274 (Mo-Kα)
Radiation wavelength, Å	0.71073	0.71073
*T*_min_/*T*_max_	0.622/0.747	0.680/0.737
*θ_min_*–*θ_max_*, deg	1.408–30.000	2.503–26.000
*F*(000)	1004	619
*R*_int_	0.0476	0.0669
Reflections measured	61,178	15,198
Independent reflections	11,829	4594
Observed reflections *I* > 2*σ*(*I*)	9870	3377
*R*_1_, *wR*_2_ (*I* > 2*σ*(*I*))	0.0430, 0.0785	0.0543, 0.1036
*GooF*	1.111	1.033
Δρ_min_/Δρ_max_, (e/Å^3^)	0.419/−0.449	1.141/−0.539

## Data Availability

The original contributions presented in this study are included in the article/Supplementary Material. Further inquiries can be directed to the corresponding author(s).

## Supplementary Materials

The following supporting information can be downloaded at https://www.mdpi.com/article/10.3390/molecules30224367/s1, Figure S1: ATR FT-IR spectra for **1**; Figure S2: ATR FT-IR spectra for **2**; Figure S3: The experimental (blue curve) and calculated (red curve) powder patterns for **1** and their difference (gray curve). Blue ticks indicated calculated positions of refined structure; Figure S4: The experimental (blue curve) and calculated (red curve) powder patterns for **2** and their difference (gray curve). Blue ticks indicated calculated positions of refined structure; Figure S5: The experimental (blue curve) and calculated (red curve) powder patterns for **3** and their difference (gray curve). Blue ticks indicated calculated positions of refined structure; Table S1. Selected bond angles (ω) in the structure of compounds **1** and **2**; Table S2. Analysis of π-π interactions in the crystal packaging of compounds **1** and **2**; Figure S6. UV–visible absorption spectra of complex **1** at various molar concentrations, measured in a 1% aqueous DMSO solution; Figure S7. UV–visible absorption spectra of complex **2** at various molar concentrations, measured in a 1% aqueous DMSO solution; Figure S8. UV–visible absorption spectra of complex **3** at various molar concentrations, measured in a 1% aqueous DMSO solution.
