# Supplementary material for: Antiproliferative Potential of Cobalt(II) Phenanthroline Complexes with Pyridonates
Source: Molecules. 2025 Nov 12;30(22):4367. doi: 10.3390/molecules30224367 (PMC12655206; doi:10.3390/molecules30224367)

checkCIF/PLATON report

Structure factors have been supplied for datablock(s) mn33_24

THIS REPORT IS FOR GUIDANCE ONLY. IF USED AS PART OF A REVIEW PROCEDURE FOR PUBLICATION, IT SHOULD NOT REPLACE THE EXPERTISE OF AN EXPERIENCED CRYSTALLOGRAPHIC REFEREE.

No syntax errors found.   CIF dictionary   Interpreting this report

Datablock: mn33_24

```
Bond precision:   C-C = 0.0031 A            Wavelength=0.71073

Cell:          a=9.3330(5)         b=14.7266(7)       c=15.0683(7)
               alpha=82.8863(16)   beta=89.2581(17)   gamma=81.6392(16)
Temperature:   100 K

                 Calculated                    Reported
Volume           2033.23(17)                   2033.23(17)
Space group      P -1                          P -1
Hall group       -P 1                          -P 1
Moiety formula   C22 H14 Cl2 Co N4 O2          C22 H14 Cl2 Co N4 O2
Sum formula      C22 H14 Cl2 Co N4 O2          C22 H14 Cl2 Co N4 O2
Mr               496.20                        496.20
Dx,g cm-3        1.621                         1.621
Z                4                             4
Mu (mm-1)        1.135                         1.135
F000             1004.0                        1004.0
F000'            1006.87
h,k,lmax         13,20,21                      13,20,21
Nref             11839                         11829
Tmin,Tmax        0.826,0.913                   0.622,0.747
Tmin'            0.815

Correction method= # Reported T Limits: Tmin=0.622 Tmax=0.747
AbsCorr = MULTI-SCAN
```

```
Data completeness= 0.999            Theta(max)= 30.000

R(reflections)= 0.0430( 9870)                              wR2(reflections)=
                                                           0.0832( 11829)
S = 1.111                  Npar= 559
```

The following ALERTS were generated. Each ALERT has the format
test-name_ALERT_alert-type_alert-level.
Click on the hyperlinks for more details of the test.

Alert level C

```
PLAT906_ALERT_3_C Large K Value in the Analysis of Variance ......      3.288 Check
PLAT911_ALERT_3_C Missing FCF Refl Between Thmin & STh/L=    0.600          8 Report
                1  0  0,   0  2  0,   0  1  1,  -2  2  1,   2  3  1,   0 -1  2,
                0  0  4,   7 10  6,
```

Alert level G

```
PLAT232_ALERT_2_G Hirshfeld Test Diff (M-X)  Co1A     --O1A      .        5.8 s.u.
PLAT232_ALERT_2_G Hirshfeld Test Diff (M-X)  Co1A     --O2A      .        7.0 s.u.
PLAT232_ALERT_2_G Hirshfeld Test Diff (M-X)  Co1B     --O1B      .        5.6 s.u.
PLAT232_ALERT_2_G Hirshfeld Test Diff (M-X)  Co1B     --O2B      .        6.4 s.u.
PLAT434_ALERT_2_G Short Inter HL..HL Contact Cl2B     ..Cl2B     .       3.19 Ang.
                                                1-x,-y,1-z  =      2_656 Check
PLAT910_ALERT_3_G Missing FCF Reflection(s) Below Theta(Min)[Deg]=       1.41 Note
                0  0  1,
PLAT912_ALERT_4_G Missing # of FCF Reflections Above STh/L=  0.600          1 Note
PLAT913_ALERT_3_G Missing # of Very Strong Reflections in FCF ....          3 Note
               -2  2  1,   2  3  1,   0  0  4,
PLAT933_ALERT_2_G Number of HKL-OMIT Records in Embedded .res File          3 Note
               -2  2  1,   0  1  1,   1  0  0,
PLAT960_ALERT_3_G Number of Intensities with I < - 2*Sigma(I) ....          2 Check
PLAT967_ALERT_5_G Note: Two-Theta Cutoff Value in Embedded .res ..       60.0 Degree
PLAT969_ALERT_5_G The 'Henn et al.' R-Factor-gap value ..........      2.494 Note
              Predicted wR2: Based on SigI**2  3.34 or SHELX Weight  7.49
PLAT978_ALERT_2_G Number C-C Bonds with Positive Residual Density.         12 Info
```

---

0 **ALERT level A** = Most likely a serious problem - resolve or explain
0 **ALERT level B** = A potentially serious problem, consider carefully
2 **ALERT level C** = Check. Ensure it is not caused by an omission or oversight
13 **ALERT level G** = General information/check it is not something unexpected

0 ALERT type 1 CIF construction/syntax error, inconsistent or missing data
7 ALERT type 2 Indicator that the structure model may be wrong or deficient
5 ALERT type 3 Indicator that the structure quality may be low
1 ALERT type 4 Improvement, methodology, query or suggestion
2 ALERT type 5 Informative message, check

It is advisable to attempt to resolve as many as possible of the alerts in all categories. Often the minor alerts point to easily fixed oversights, errors and omissions in your CIF or refinement strategy, so attention to these fine details can be worthwhile. It is up to the individual to critically assess their own results and, if necessary, seek expert advice.

PLATON version of 04/06/2025; check.def file version of 30/05/2025

---

duplicate check

No duplication found

Datablock mn33_24 - ellipsoid plot

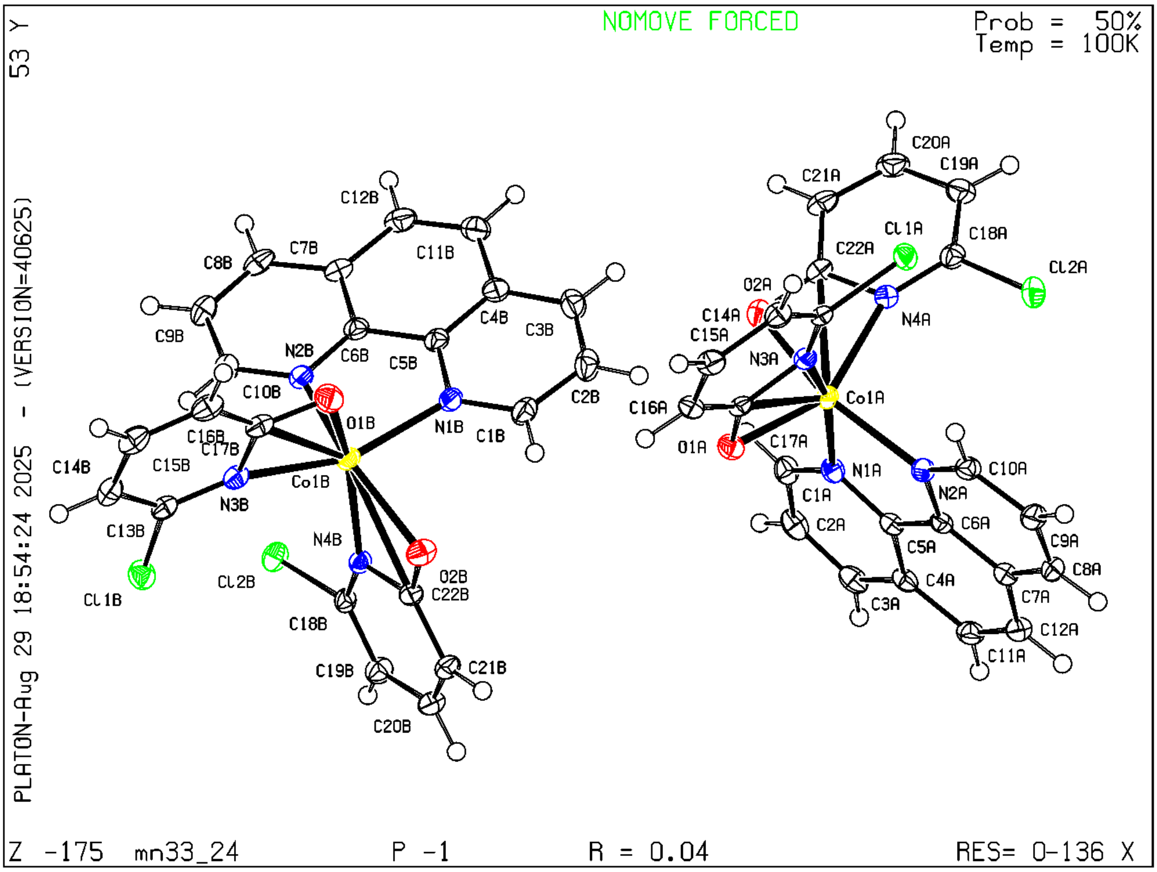

Supplement: Supplementary file 1 [file molecules-30-04367-s001.zip › checkcif_1_2483770.pdf]
