# Supplementary material for: Antiproliferative Potential of Cobalt(II) Phenanthroline Complexes with Pyridonates
Source: Molecules. 2025 Nov 12;30(22):4367. doi: 10.3390/molecules30224367 (PMC12655206; doi:10.3390/molecules30224367)

checkCIF/PLATON report

Structure factors have been supplied for datablock(s) 37_24p

THIS REPORT IS FOR GUIDANCE ONLY. IF USED AS PART OF A REVIEW PROCEDURE FOR PUBLICATION, IT SHOULD NOT REPLACE THE EXPERTISE OF AN EXPERIENCED CRYSTALLOGRAPHIC REFEREE.

No syntax errors found.   CIF dictionary   Interpreting this report

Datablock: 37_24p

```
Bond precision:   C-C = 0.0071 A          Wavelength=0.71073

Cell:            a=9.7217(8)        b=11.0191(9)      c=12.0031(11)
                 alpha=69.720(3)    beta=76.610(3)    gamma=87.115(3)
Temperature:     100 K

                 Calculated                   Reported
Volume           1172.70(18)                  1172.70(18)
Space group      P -1                         P -1
Hall group       -P 1                         -P 1
Moiety formula   C42 H22 Cl2 Co3 F12 N6 O10  C42 H22 Cl2 Co3 F12 N6 O10
Sum formula      C42 H22 Cl2 Co3 F12 N6 O10  C42 H22 Cl2 Co3 F12 N6 O10
Mr               1246.35                      1246.34
Dx,g cm-3        1.765                        1.765
Z                1                            1
Mu (mm-1)        1.274                        1.274
F000             619.0                        619.0
F000'            620.73
h,k,lmax         11,13,14                     11,13,14
Nref             4609                         4594
Tmin,Tmax        0.837,0.926                  0.680,0.737
Tmin'            0.837

Correction method= # Reported T Limits: Tmin=0.680 Tmax=0.737
AbsCorr = MULTI-SCAN
```

Data completeness= 0.997 Theta(max)= 26.000

R(reflections)= 0.0543(3377) wR2(reflections)= 0.1218(4594)

S = 1.033 Npar= 353

The following ALERTS were generated. Each ALERT has the format
test-name_ALERT_alert-type_alert-level.
Click on the hyperlinks for more details of the test.

Alert level C

```
PLAT094_ALERT_2_C Ratio of Maximum / Minimum Residual Density ....       2.12 Report
PLAT213_ALERT_2_C Atom F3               has ADP max/min Ratio .....       3.2 prolat
PLAT213_ALERT_2_C Atom F4               has ADP max/min Ratio .....       3.1 prolat
PLAT213_ALERT_2_C Atom F4'              has ADP max/min Ratio .....       3.1 prolat
PLAT234_ALERT_4_C Large Hirshfeld Difference F6       --C21       .      0.16 Ang.
PLAT341_ALERT_3_C Low Bond Precision on  C-C Bonds ..............    0.00712 Ang.
PLAT601_ALERT_2_C Unit Cell Contains Solvent Accessible VOIDS   <=        72 Ang**3
PLAT911_ALERT_3_C Missing FCF Refl Between Thmin & STh/L=    0.600        11 Report
                9  3  0,  -7 -7  1,   8  6  1,   2  0  2,  -7  3  2,   9  4  2,
                4 -7  4,  -1 -4  9,  -6  0  9,   0  0 11,  -1  1 11,
```

Alert level G

```
PLAT002_ALERT_2_G Number of Distance or Angle Restraints on AtSite          9 Note
PLAT003_ALERT_2_G Number of Uiso or U(i,j)  Restrained non-H-Atoms          4 Report
PLAT154_ALERT_1_G The s.u.'s on the Cell Angles are Equal ..(Note)      0.003 Degree
PLAT171_ALERT_4_G The CIF-Embedded .res File Contains EADP Records          4 Report
PLAT172_ALERT_4_G The CIF-Embedded .res File Contains DFIX Records          2 Report
PLAT186_ALERT_4_G The CIF-Embedded .res File Contains ISOR Records          2 Report
PLAT242_ALERT_2_G Low    'MainMol' Ueq as Compared to Neighbors of        C19 Check
PLAT301_ALERT_3_G Main Residue  Disorder ..............(Resd    1)        11% Note
PLAT434_ALERT_2_G Short Inter HL..HL Contact Cl1       ..F3       .      2.97 Ang.
                                                   x,1+y,z  =      1_565 Check
PLAT794_ALERT_5_G Tentative Bond Valency for Co2       (II)       .      2.11 Info
PLAT860_ALERT_3_G Number of Least-Squares Restraints ............          31 Note
PLAT883_ALERT_1_G Absent Datum for _atom_sites_solution_primary ..    Please Do !
PLAT910_ALERT_3_G Missing FCF Reflection(s) Below Theta(Min)[Deg]=       2.50 Note
                1  0  0,   0  1  0,   0  0  1,   0  1  1,
PLAT933_ALERT_2_G Number of HKL-OMIT Records in Embedded .res File          3 Note
                0  1  0,   0  1  1,  -7  3  2,
PLAT941_ALERT_3_G Average HKL Measurement Multiplicity ..........         3.3 Low
PLAT960_ALERT_3_G Number of Intensities with I < - 2*Sigma(I) ....          5 Check
PLAT967_ALERT_5_G Note: Two-Theta Cutoff Value in Embedded .res ..       52.0 Degree
PLAT969_ALERT_5_G The 'Henn et al.' R-Factor-gap value ..........       2.117 Note
              Predicted wR2: Based on SigI**2  5.75 or SHELX Weight 11.78
PLAT978_ALERT_2_G Number C-C Bonds with Positive Residual Density.          2 Info
```

---

0 **ALERT level A** = Most likely a serious problem - resolve or explain
0 **ALERT level B** = A potentially serious problem, consider carefully
8 **ALERT level C** = Check. Ensure it is not caused by an omission or oversight
19 **ALERT level G** = General information/check it is not something unexpected

```
 2 ALERT type 1 CIF construction/syntax error, inconsistent or missing data
11 ALERT type 2 Indicator that the structure model may be wrong or deficient
 7 ALERT type 3 Indicator that the structure quality may be low
 4 ALERT type 4 Improvement, methodology, query or suggestion
 3 ALERT type 5 Informative message, check
```

It is advisable to attempt to resolve as many as possible of the alerts in all categories. Often the minor alerts point to easily fixed oversights, errors and omissions in your CIF or refinement strategy, so attention to these fine details can be worthwhile. It is up to the individual to critically assess their own results and, if necessary, seek expert advice.

PLATON version of 04/06/2025; check.def file version of 30/05/2025

---

duplicate check

No duplication found

Datablock 37_24p - ellipsoid plot

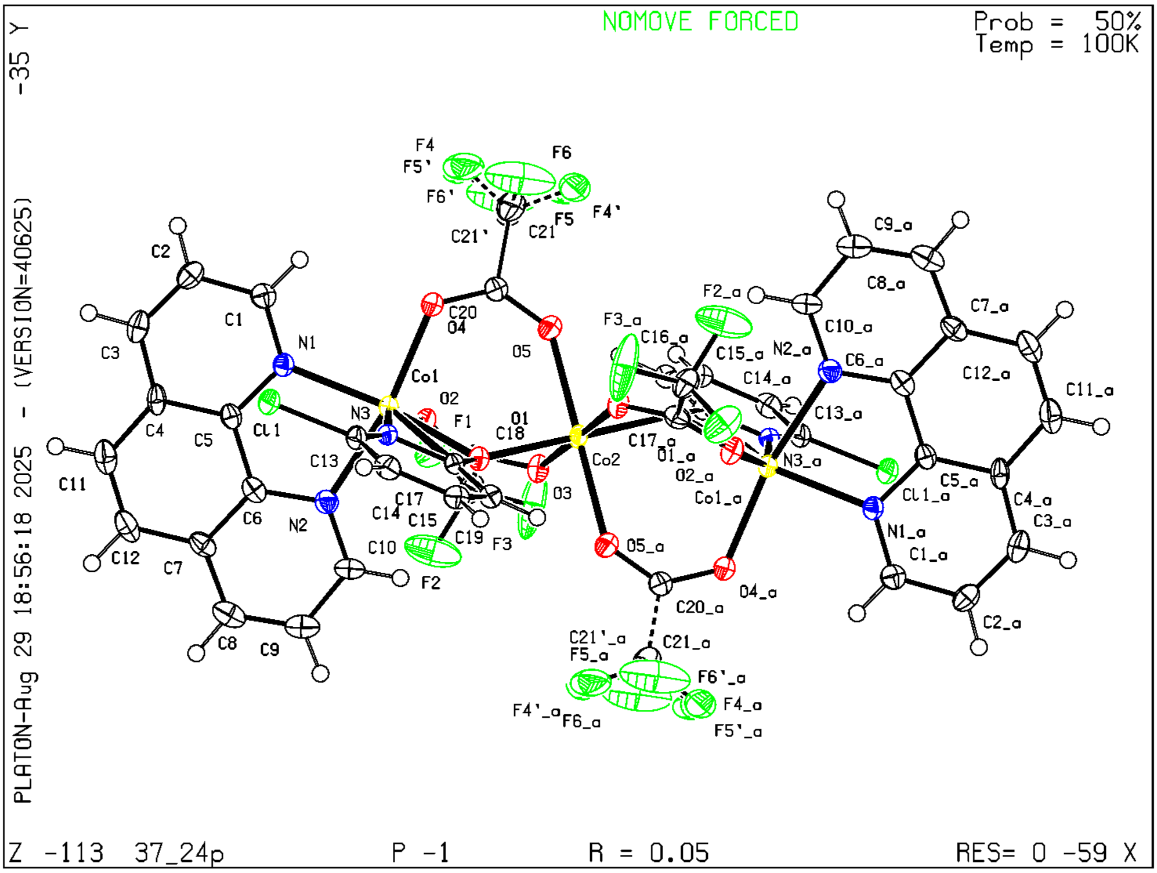

Supplement: Supplementary file 1 [file molecules-30-04367-s001.zip › checkcif_22483771.pdf]
