# Supplementary material for: Antiproliferative Potential of Cobalt(II) Phenanthroline Complexes with Pyridonates
Source: Molecules. 2025 Nov 12;30(22):4367. doi: 10.3390/molecules30224367 (PMC12655206; doi:10.3390/molecules30224367)
Supplement: Supplementary file 1 [file molecules-30-04367-s001.zip › molecules-3955403-supplementary.pdf]

molecules

Article

Antiproliferative Potential of Cobalt(II) Phenanthroline Complexes with Pyridonates

Marina E. Nikiforova [1,*], Irina A. Lutsenko[1,2,*], Fedor M. Dolgushin [1], Maxim A. Shmelev [1], Alexey A. Sidorov [1], Dmitriy S. Yambulatov [1], Darina A. Sokolova [2,3], Vadim S. Pokrovsky [2,3] and Igor L. Eremenko [1]

1 N.S. Kurnakov Institute of General and Inorganic Chemistry, Russian Academy of Sciences, 119991 Moscow, Russia; fmdolgushin@gmail.com (F.M.D.); shmelevma@yandex.ru (M.A.S.); sidorov@igic.ras.ru (A.A.S.); yambulatov@yandex.ru (D.S.Y.); ilerem@igic.ras.ru (I.L.E.)
2 Patrice Lumumba Peoples' Friendship University, 117198, Moscow, Russia; d.v.sokolova@gmail.com (D.V.S.); pokrovskiy-vs@rudn.ru (V.S.P.)
3 N.N. Blokhin National Medical Research Center of Oncology, 115478, Moscow, Russia
* Correspondence: nikiforova.marina@gmail.com (M.E.N.); irinalu05@rambler.ru (I.A.L.)

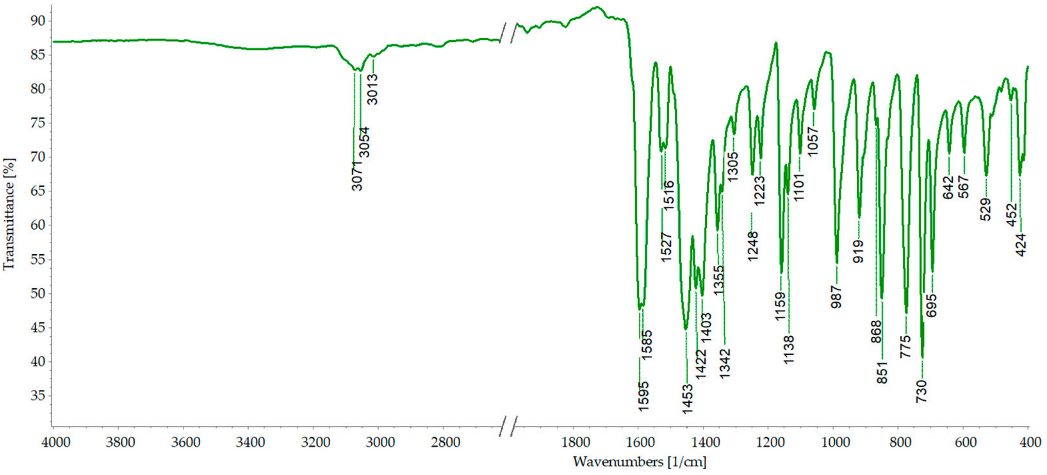

Figure S1. ATR FT-IR spectra for **1**.

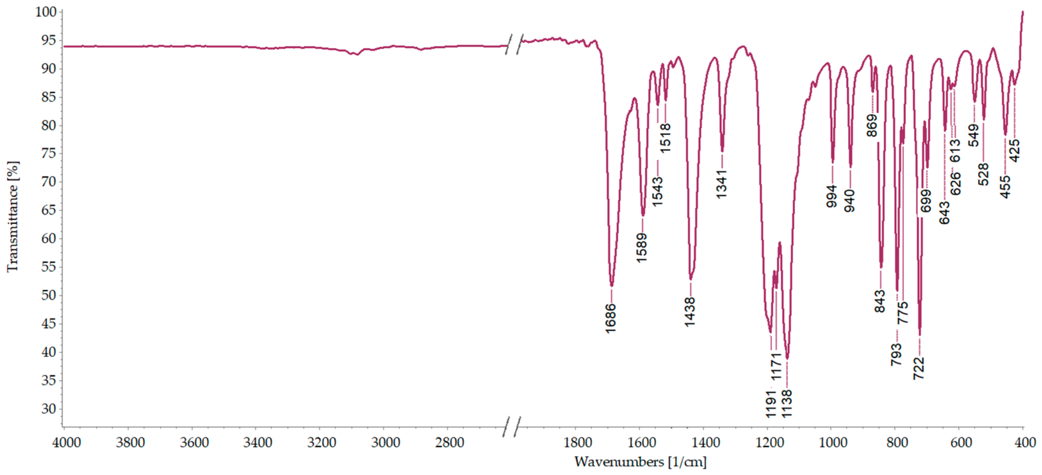

Figure S2. ATR FT-IR spectra for **2**.

 https://doi.org/10.3390/molecules30224367

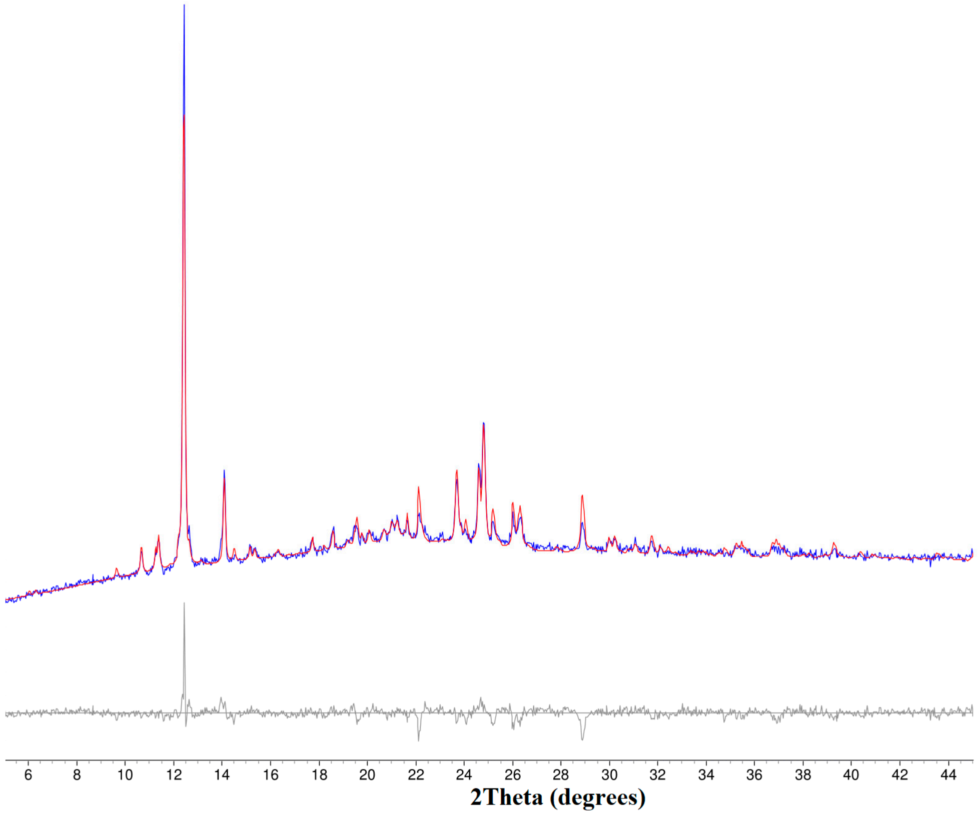

Figure S3. The experimental (blue curve) and calculated (red curve) powder patterns for **1** and their difference (grey curve). Blue ticks indicated calculated positions of refined structure.

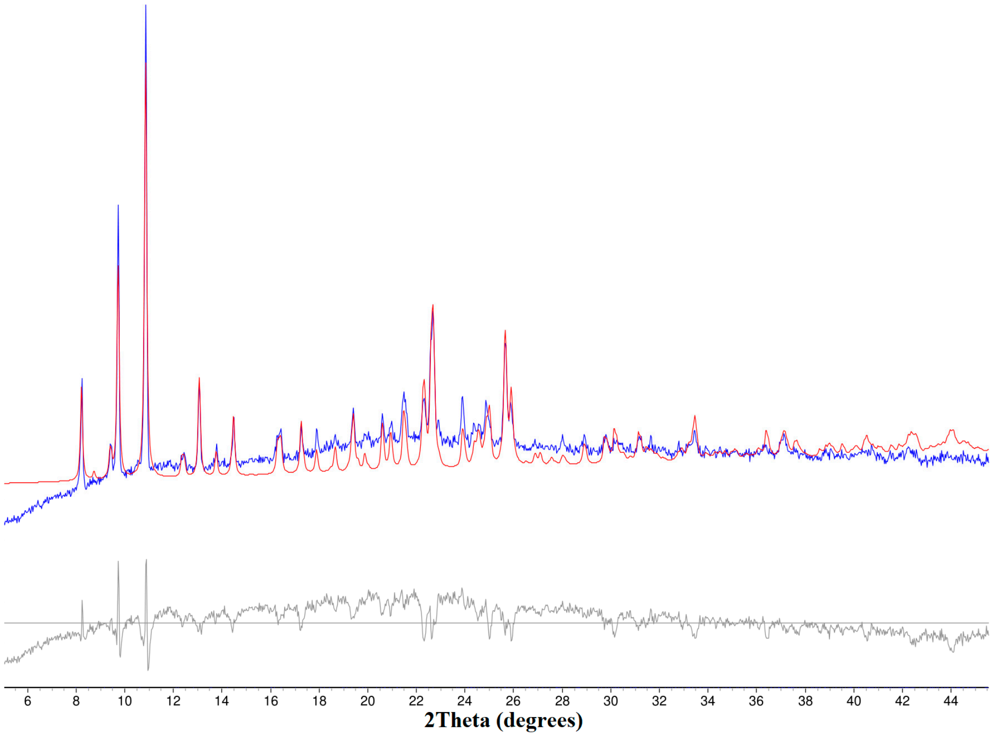

Figure S4. The experimental (blue curve) and calculated (red curve) powder patterns for **2** and their difference (grey curve). Blue ticks indicated calculated positions of refined structure.

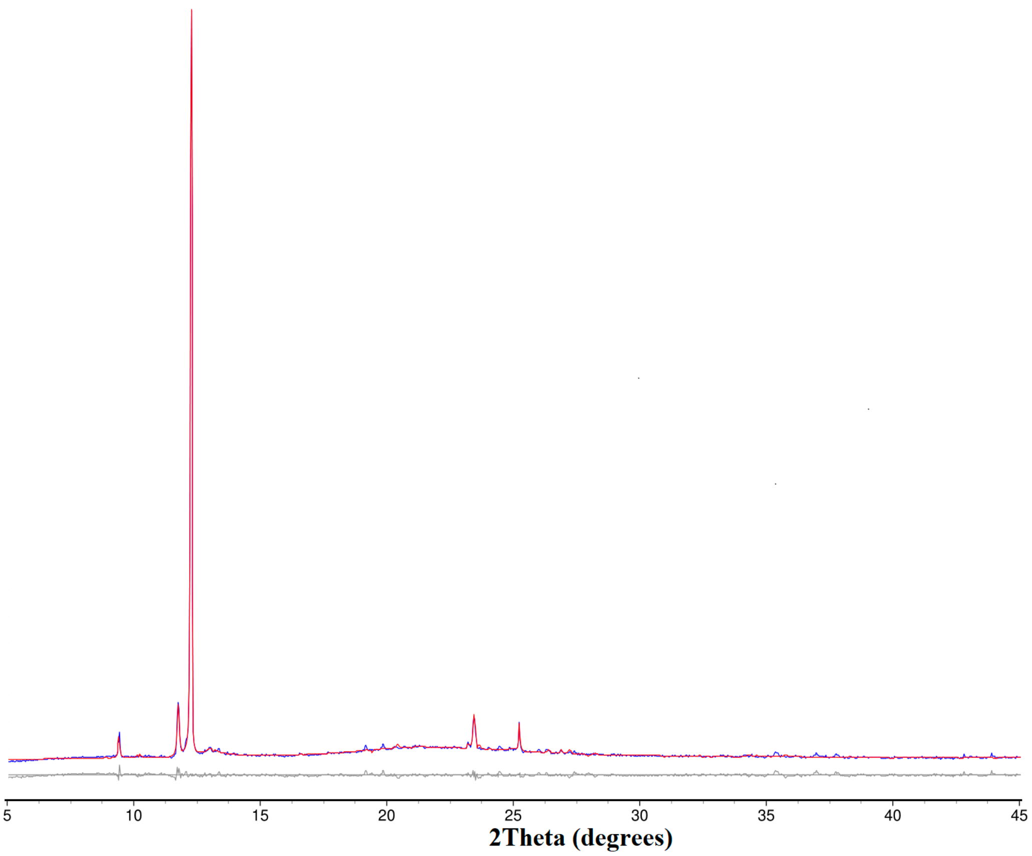

Figure S5. The experimental (blue curve) and calculated (red curve) powder patterns for **3** and their difference (grey curve). Blue ticks indicated calculated positions of refined structure.

Table S1. Selected bond angles (ω) in the structure of compounds **1** and **2**.

**1**		**2**	
Angle	ω, deg	Angle	ω, deg
O1A—Co1A—O2A	95.42(6)	O1 Co1 N2	97.00(13)
O1A—Co1A—N1A	100.49(6)	O1 Co1 N3	61.17(12)
O1A—Co1A—N2A	105.06(6)	O2 Co1 O1	93.20(12)
O1A—Co1A—N3A	62.78(6)	O2 Co1 N1	108.10(13)
O1A—Co1A—N4A	149.51(6)	O2 Co1 N2	83.85(14)
O2A—Co1A—N3A	101.56(6)	O2 Co1 N3	152.32(13)
N1A—Co1A—O2A	89.33(6)	O4 Co1 O1	94.70(12)
N1A—Co1A—N3A	160.54(7)	O4 Co1 O2	93.40(12)
N1A—Co1A—N4A	100.06(7)	O4 Co1 N1	91.49(13)
N2A—Co1A—O2A	157.85(6)	O4 Co1 N2	168.11(14)
N2A—Co1A—N1A	78.86(7)	O4 Co1 N3	98.69(13)
N2A—Co1A—N3A	95.34(6)	N1 Co1 O1	157.42(13)
N2A—Co1A—N4A	100.83(7)	N1 Co1 N2	78.46(14)
N4A—Co1A—O2A	62.57(6)	N1 Co1 N3	96.44(14)
N4A—Co1A—N3A	99.27(7)	N2 Co1 N3	88.83(14)
O1B—Co1B—O2B	98.26(6)	O1 Co2 O1	180.0
O1B—Co1B—N1B	98.69(7)	O1 Co2 O3	92.59(12)
O1B—Co1B—N2B	100.15(7)	O1 Co2 O3	87.41(12)
O1B—Co1B—N3B	63.05(6)	O1 Co2 O3	87.41(12)

O1B—Co1B—N4B	153.64(7)	O1 Co2 O3	92.59(12)
O2B—Co1B—N3B	103.72(6)	O1 Co2 O5	92.21(12)
N1B—Co1B—O2B	89.26(6)	O1 Co2 O5	87.79(12)
N1B—Co1B—N2B	78.70(7)	O1 Co2 O5	92.21(12)
N1B—Co1B—N3B	158.61(7)	O1 Co2 O5	87.79(12)
N1B—Co1B—N4B	98.74(7)	O3 Co2 O3	180.0
N2B—Co1B—O2B	159.29(7)	O3 Co2 O5	89.51(12)
N2B—Co1B—N3B	93.05(7)	O3 Co2 O5	90.49(12)
N4B—Co1B—O2B	62.39(6)	O3 Co2 O5	89.51(12)
N4B—Co1B—N2B	102.54(7)	O3 Co2 O5	90.49(12)
N4B—Co1B—N3B	102.32(7)	O5 Co2 O5	180.0

Table S2. Analysis of π-π interactions in the crystal packaging of compounds **1** and **2**.

Interactions	Symmetry code	Cg···Cg, Å	Cg···Perp	α, deg	β, deg	γ, deg
Compound **1**						
phen(N1A->C5A)···phen(N1A->C5A)	-X,1-Y,-Z	3.6638(12)	3.3325(8)	0.00(9)	24.6	24.6
phen(N1A->C5A)···phen(C4A->C11A)	-X,1-Y,-Z	3.5188(12)	3.3442(8)	2.37(9)	16.6	18.1
phen(C4A->C11A)···phen(N1A->C5A)	-X,1-Y,-Z	3.5187(12)	3.3719(8)	2.37(9)	18.1	16.6
phen(N1B->C5B)···phen(C4B->C11B)	1-X,1-Y,1-Z	3.5368(13)	3.3923(9)	1.65(10)	17.1	16.4
phen(C4B->C11B)···phen(N1B->C5B)	1-X,1-Y,1-Z	3.5369(13)	3.3809(9)	1.65(10)	16.4	17.1
Compound **2**						
phen(N1->C5)···phen(C4->C11)	1-X,1-Y,-Z	3.547(3)	3.448(2)	2.2(2)	11.4	13.6
phen(N2->C10)···phen(N2->C10)	-X,1-Y,-Z	3.587(3)	3.258(2)	0.0(2)	24.7	24.7
phen(C4->C11)···phen(N1->C5)	1-X,1-Y,-Z	3.548(3)	3.478(2)	2.2(2)	13.6	11.4

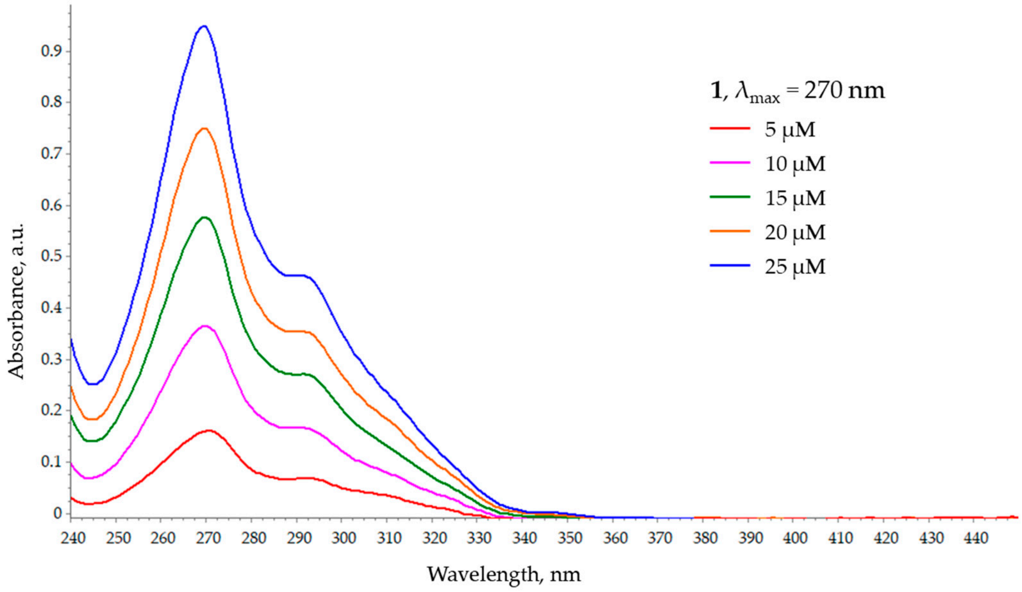

Figure S6. UV-visible absorption spectra of complex **1** at various molar concentrations, measured in a 1% aqueous DMSO solution.

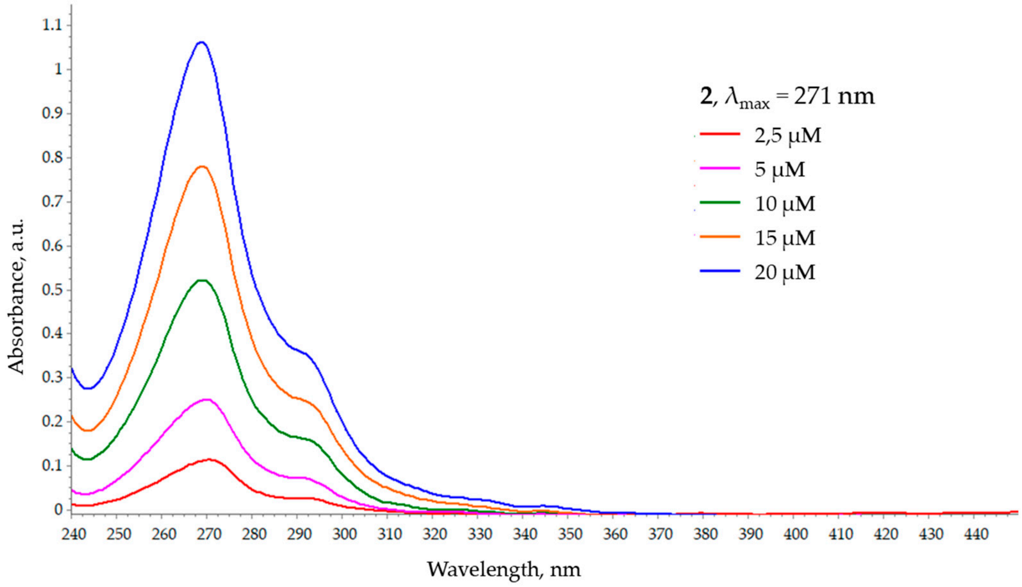

Figure S7. UV-visible absorption spectra of complex **2** at various molar concentrations, measured in a 1% aqueous DMSO solution.

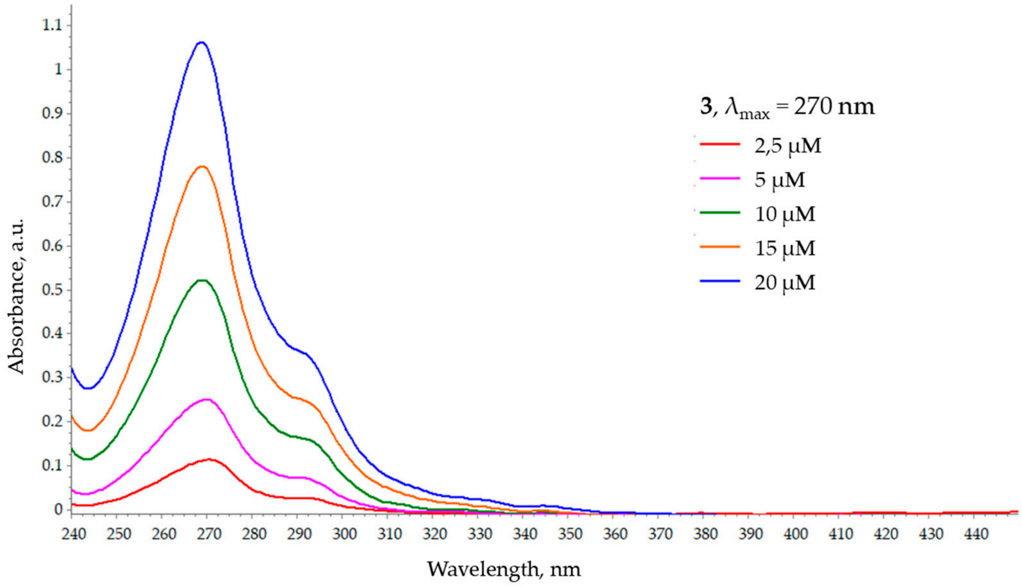

Figure S8. UV-visible absorption spectra of complex **3** at various molar concentrations, measured in a 1% aqueous DMSO solution.
